# Evaluating the Impact of Common Non-Oncologic Medication Use During Radiotherapy in Patients with High-Risk Prostate Cancer

**DOI:** 10.3390/curroncol32060353

**Published:** 2025-06-15

**Authors:** Haley K. Perlow, Karishma Khullar, Ritesh Kumar, Sonya Sasmal, Kent Nakamoto, Yevgeniya Gokun, Jacob Eckstein, Rebekah Young, Dayssy A. Diaz, Douglas Martin, Katharine A. Collier, Lingbin Meng, Rahul R. Parikh, Steven Clinton, Shang-Jui Wang

**Affiliations:** 1 Department of Radiation Oncology, The Ohio State University College of Medicine and Comprehensive Cancer Center, 460 W. 10th Avenue, Columbus, OH 43210, USA; haley.perlow@uhhospitals.org (H.K.P.); jacob.eckstein@osumc.edu (J.E.); rebekah.young@osumc.edu (R.Y.); dayssy.diazpardo@osumc.edu (D.A.D.); douglas.martin@osumc.edu (D.M.); 2 Department of Radiation Oncology, University Hospitals Seidman Cancer Center, Case Western Reserve School of Medicine, Cleveland, OH 44106, USA; 3 Department of Radiation Oncology, Perelman School of Medicine, University of Pennsylvania, Philadelphia, PA 19104, USA; khullar.karishma@gmail.com; 4 Department of Radiation Oncology, Rutgers Cancer Institute of New Jersey, New Brunswick, NJ 08901, USA; rk912@cinj.rutgers.edu (R.K.); parikhrr@cinj.rutgers.edu (R.R.P.); 5 College of Medicine, The Ohio State University, Columbus, OH 43210, USA; sonya.sasmal@osumc.edu (S.S.); kent.nakamoto@osumc.edu (K.N.); 6 Center for Biostatistics, The Ohio State University, Columbus, OH 43210, USA; yevgeniya.gokun@osumc.edu; 7 Division of Medical Oncology, Department of Internal Medicine, The Ohio State University College of Medicine and Comprehensive Cancer Center, Columbus, OH 43210, USA; katharine.collier@osumc.edu (K.A.C.); lingbin.meng@osumc.edu (L.M.); steven.clinton@osumc.edu (S.C.)

**Keywords:** prostate cancer, radiotherapy, metformin, aspirin, statin

## Abstract

Introduction: The treatment efficacy of prostate cancer (PCa) radiotherapy (RT) can be inadvertently affected by the concurrent usage of non-oncologic medications. Many studies have associated the intake of several non-oncologic drugs with cancer specific outcomes. In this study, we report the impact of daily non-oncologic medications including aspirin, metformin, and statins on time to progression for patients with high-risk PCa. Methods: Patients with high- and very high risk PCa (NCCN definition of Gleason score ≥ 8, prostate-specific antigen (PSA) ≥ 20, or ≥cT3a) who received definitive RT at two institutions were included in this analysis. Progression was defined as either biochemical (PSA > nadir + 2 ng/mL), locoregional (prostate or lymph nodes, biopsy-proven), or development of distant metastases. Progression-free survival (PFS) was defined as the time elapsed from the start of RT to progression or last follow-up. Cox proportional hazards models evaluated the associations between non-oncologic medications and PFS. Results: There were 237 patients eligible for this analysis, of which 47 (19.8%) and 178 (75.1%) had at least clinical T3 disease or at least Gleason 8 disease, respectively. During RT, 82 (34.6%), 88 (37.1%), and 29 (12.2%) patients were taking aspirin, statin, or metformin, respectively. Overall, 54 patients (22.8%) experienced disease progression. Neither aspirin nor statin usage had a significant association with PFS. Patients prescribed metformin displayed worse PFS compared to patients not taking metformin (aHR: 2.46, 95% CI: 1.06–5.72). Conclusions: Aspirin and statin usage was not associated with likelihood of progression in this large cohort of patients with high-/very high risk PCa. Metformin use was associated with poorer PFS, albeit with a small event rate due to fewer patients taking metformin. Further studies are needed to clarify the impact of common non-oncologic medication use on outcomes for patients with high-risk PCa.

## 1. Introduction

The concurrent use of non-oncologic medications has the potential to impact prostate cancer treatments. Preclinical studies have suggested that several common medications, such as aspirin, metformin, and statins, show potential as therapeutic strategies against prostate cancer. Aspirin, which functions as a cyclooxygenase-2 inhibitor, has been shown to have antitumor activities against prostate cancer through the actions of apoptosis and the inhibition of angiogenesis [1]. Metformin has been shown to reduce androgen receptor protein expression and its pathway signaling, which regulates proliferation, invasion, differentiation, and apoptosis of prostate cancer [2,3,4]. Statins can trigger apoptosis of prostate cancer cells through HMG-CoA reductase inhibition [5], and prostate cancer patients receiving neoadjuvant statin therapy prior to prostatectomy demonstrated reduced tumor proliferation and increased apoptosis in the surgical prostate specimen [6,7]. Despite promising preclinical results, clinical outcome data of aspirin/metformin/statins use in prostate cancer patients are mixed, at best, and the benefit of these non-oncologic medications for the treatment or prevention of prostate cancer remains indeterminate [2,8,9,10,11,12,13]. These studies are mostly population-based epidemiological studies with vast heterogeneity in patient characteristics, disease spectrum, and treatment modality, which underscores the further need to dissect the effect of these medications in more specific homogenous subsets of prostate cancer patients. In this study, we aim to retrospectively examine the impact of three common non-oncologic medications (aspirin, metformin, and statins) in National Comprehensive Cancer Network (NCCN) high-risk prostate cancer patients treated with definitive radiotherapy.

## 2. Methods

This retrospective study was approved by the institutional review board at two participating institutions. Patients treated from 2005 to 2017 were analyzed. Patients with high-risk (inclusive of very high risk) prostate adenocarcinoma were eligible for inclusion; high risk was defined as a T stage of 3a or higher, a Gleason score of 8 or higher, or PSA > 20. Eligible patients who received external beam radiotherapy, with or without a brachytherapy boost, were identified and analyzed at both institutions. Diagnostic workup and radiation treatment for each patient were not required to be performed at the same hospital. Relevant demographic and clinical information were collected for each patient.

Medical documentation was retrospectively analyzed, and prescription of non-oncologic medications (aspirin, metformin, and statin) at the time of radiation oncology consultation was documented. Given that aspirin, metformin, and statin are medications intended for long-term use for cardioprotection, glycemic control, and cholesterol reduction, respectively, it is presumed that the prescription of these medications reflects a regular daily usage.

Progression-free survival (PFS) was defined as the date from radiotherapy initiation to biochemical recurrence, locoregional recurrence, or distant recurrence. Biochemical progression was defined as PSA rising to the nadir + 2 ng/mL after radiotherapy conclusion. Locoregional recurrence was defined as radiographic or biopsy-proven recurrence in the prostate or regional lymph nodes. Distant recurrence was defined as radiographic or biopsy-proven recurrence outside the prostate or regional lymph nodes. Labs or imaging was required to evaluate for recurrent disease. Patients were censored by date of death or last follow-up.

### Statistics

Descriptive statistics such as medians and interquartile ranges (IQRs) were used for continuous variables, while frequencies and percents were used for categorical variables. Kaplan–Meier curves along with log-rank tests were used to evaluate the time to progression for users and non-users of aspirin, statins, or metformin. Univariate Cox proportional hazards regressions evaluated the associations between demographic and clinical characteristics, including aspirin, statin, and metformin prescriptions and PFS. For the main analysis, three separate multivariable Cox proportional hazards regressions were implemented (one for each medication prescription), adjusting for variables that were clinically meaningful or statistically significant in the unadjusted results (PSA at diagnosis, T stage, Gleason score, and race/ethnicity). We further investigated the statistical significance of metformin use by running two additional models as part of our sensitivity analysis—one with adjustment for PSA at diagnosis, T stage, Gleason score, and obesity (body mass index [BMI] ≥ 30), and the other one with adjustment for PSA at diagnosis, T stage, Gleason score, and ADT duration (<6 months vs. ≥6 months). These analyses were performed using SAS v9.4 (SAS Institute; Cary, NC, USA). Statistical significance was defined as two-sided alpha of 0.05.

## 3. Results

A total of 237 patients were included in this analysis (Table 1). The median follow-up for the study cohort was 3.5 years. The median age at prostate cancer diagnosis was 66 years (range 43–87). The median PSA at diagnosis was 16.2 (IQR: 7.7–35.0). Overall, 19.8% of the patients had T3 or higher stage disease, and 38.1%, 33.1%, and 4.2% of the patients had Gleason 8, Gleason 9, and Gleason 10 disease, respectively. All patients received intensity-modulated radiation therapy (IMRT), with 93.7% receiving dose-escalated IMRT, and 6.3% receiving IMRT with brachytherapy boost. For patients who received dose-escalated IMRT, the median dose was 78 Gy (interquartile range 77.4–79.2 Gy). Almost all patients (94.5%) received androgen deprivation therapy (ADT) with median duration of 20 months (interquartile range 9–24 months). Regular aspirin, statin, or metformin use was identified in 34.6%, 37.1%, and 12.2% of the patients, respectively, at the time of radiation oncology consultation.

**Figure 1:**
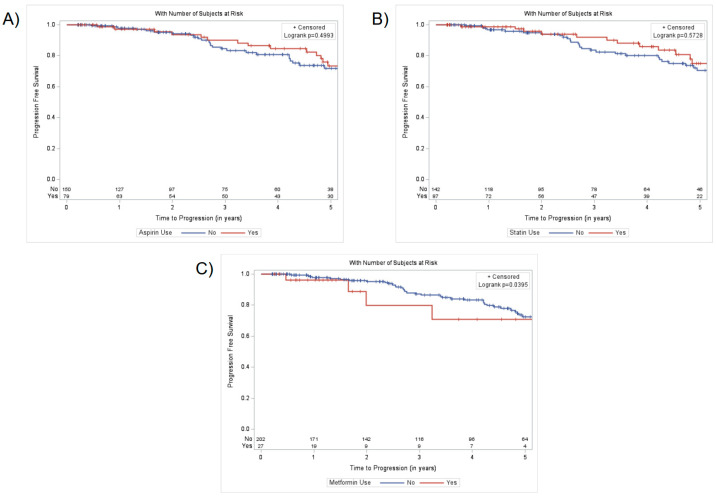
Association between progression-free survival of high-risk prostate cancer patients treated with definitive radiotherapy and (**A**) aspirin use, (**B**) statin use, and (**C**) metformin use.

Using the Kaplan–Meier method, there were no significant relationships between PFS and aspirin (*p* = 0.4993) or statin (*p* = 0.5728) use (Figure 1). The 4-year PFS for aspirin users and non-users was 85% (95% CI 72–92%) and 81% (95% CI 72–87%), respectively, and that for statin users and non-users was 86% (95% CI: 73–93%) and 80% (95% CI: 71–87%), respectively. However, metformin use was significantly associated with decreased PFS (*p* = 0.0395), with 4-year PFS for metformin users and non-users of 71% (95% CI: 38–89%) and 83% (95% CI: 76–88%), respectively.

In the univariate Cox proportional hazards model analyses, patients with PSA of ≥20 were at a 1.8-time-higher risk of progression compared to patients with PSA lower than 20 (HR: 1.81, 95% CI: 1.04–3.12). Patients who had at least T3 disease were at a 2-time-higher risk of progression compared to those with either T1 or T2 disease (HR: 2.00, 95% CI: 1.11–3.60), while patients who used metformin had a 2.2-time-higher risk of progression compared to patients who did not use metformin (HR: 2.20, 95% CI: 1.02–4.72). Neither aspirin nor statin use were significantly associated with PFS (Table 2).

Three separate multivariable Cox proportional hazard regressions evaluated PFS for users vs. non-users of aspirin, statin, or metformin, while adjusting for PSA, Gleason score, T stage, and race/ethnicity (Table 3, Table 4 and Table 5). In all three models, PSA ≥ 20, T3-T4 disease, and Gleason score ≥8 were significantly associated with cancer progression. While aspirin and statin use were not significantly associated with PFS, metformin users were at a 2.5-time-higher risk of progression compared to metformin non-users (aHR: 2.46, 95% CI: 1.06–5.72). Race/ethnicity was not significantly associated with PFS in any of the three models. Since many patients in our cohort take more than one of the three non-oncological medications, we also performed a multivariable regression analysis that included all three medications to account for possible confounding. Adjusting for PSA, Gleason score, and T stage, metformin use remained significantly associated with worse PFS (aHR: 2.77, 95% CI: 1.21–6.32), while aspirin and statin use was not significantly associated with PFS (Table 6).

Additionally, sensitivity analyses evaluating PFS for metformin users and non-users were performed with two models that adjusted for obese status and ADT duration, respectively. Given that metformin use may be confounded by the metabolic status, a multivariable regression analysis was performed with adjustment for PSA, T stage, Gleason score, and obesity (BMI ≥ 30), and metformin use remained significantly associated with worse PFS (aHR: 2.30, 95% CI: 1.01–5.22) (Table 7). Even though ADT duration was not significantly associated with PFS in the univariate analyses (Table 2), given the clinical significance of ADT duration for treatment and outcome of high-risk prostate cancer, we adjusted for this covariate in a separate model, and the association between metformin use and decreased PFS remained (aHR: 2.51, 95% CI: 1.13–5.60) (Table 8).

## 4. Discussion

This is a large retrospective analysis examining the impact of non-oncologic medications on high-risk prostate cancer outcomes. This multi-institutional analysis included 237 patients, many of whom took aspirin, statin, or metformin. Within our cohort, higher PSA, higher T stage, higher Gleason score, and metformin usage were all significant predictors of worse progression. Aspirin or statin use was not a significant predictor of our outcome.

The impact of aspirin, or COX inhibition in general, on treatment outcomes for patients with prostate cancer is mixed. This is likely due to the heterogeneity of patient populations and presentations and the limited ability to confirm adherence and frequency of COX inhibitor administration retrospectively [8]. One randomized study involving over 20,000 patients in the 1980s suggested that a regular aspirin usage can lower the risk of lethal prostate cancer both before and after diagnosis [14]. However, the STAMPEDE trial prospectively examined adding celecoxib 400 mg BID for patients with high-risk prostate cancer starting hormonal therapy and showed no benefit with the addition of COX inhibition [9]. A large meta-analysis of 10 studies showed that aspirin did not decrease the incidence of prostate cancer-specific mortality (PCSM) [10]. Multiple additional studies showed no association between aspirin use and PCSM or incidence of prostate cancer [15,16], and one study even suggested that aspirin use may increase the risk of prostate cancer [17]. In our study, COX inhibition was not associated with progression-free survival.

It has also been proposed that statin usage may impact prostate cancer outcomes. The proposed mechanisms of action include reduction in dehydroepiandrosterone sulfate uptake or reduction in androgen concentration, which may in turn improve prostate cancer progression-free survival [18,19]. Multiple retrospective studies and meta-analyses have suggested that statin usage slows the development of castrate resistance after initiation of ADT and can improve biochemical recurrence, prostate-cancer specific mortality, and overall survival [20,21,22,23]. However, the PRO-STAT randomized clinical trial evaluating adjuvant low-dose statin use after radical prostatectomy showed no difference in 1-year biochemical recurrence. Similarly, our data show no evidence of improved progression-free survival with statin usage. Larger cohorts, lengthened follow-up, and accruing higher risk patients onto clinical trials may provide a more definitive answer for the role of statin usage in improving disease outcomes.

The ability of metformin to impact prostate cancer outcomes is unclear. The presence of metabolic syndrome is shown to lead to shorter time to castrate resistance [24]. Therefore, it has been theorized that metformin, through mitigation of metabolic syndrome, can improve disease outcomes for patients with prostate cancer [2]. Clinically, the correlation of disease outcomes with metformin use is mixed, with some data suggesting an improvement of prostate cancer outcomes with the addition of metformin, some showing no benefit, and some associated with detriment [13,25,26,27]. The clinical benefit/detriment of metformin in prostate cancer is still unclear, but perhaps an alternative perspective needs to be considered, in which metformin use is a surrogate for an underlying metabolic dysregulation in the host. For example, one study showed that men with type 2 diabetes, both not on medication and on insulin, have increased prostate cancer mortality, suggesting that a poorly controlled metabolic state of diabetes may contribute to prostate cancer progression and disease aggressiveness [28]. Consistent with this hypothesis, a retrospective study showed that radiographic PFS was significantly shorter in metastatic castrate-resistant prostate cancer patients who were metformin users [25]. Interestingly, an exploratory analysis of the Metformin Active Surveillance Trial (MAST) showed that patients with obesity (BMI ≥ 30) had a worse PFS probability when randomized to metformin use [29]. Our data showed that metformin use was associated with a higher risk of prostate cancer progression in men with high-risk prostate cancer who received definitive RT/ADT. In light of the MAST study results, we also performed a sensitivity analysis that adjusted for obese status (BMI ≥ 30), but interestingly, our results showed that metformin use remained significantly associated with worse PFS. Our result is also congruent with the above hypothesis that perhaps it is the diabetic state of the patients taking metformin that drives disease progression. This possibility of confounding underscores the importance of evaluating the effect of metformin on prostate cancer outcome, either in the absence of diabetes or while controlling for the presence of diabetes. Several phase 3 clinical trials including the PRIME trial (NCT03561961) and the STAMPEDE trial Arm K (NCT00268476) are currently investigating the potential anti-neoplastic effect of metformin for men with prostate cancer.

This study retains importance for examining the association between aspirin/statin/metformin usage and clinical outcomes within a specific subset of men with prostate cancer—NCCN high-/very high risk prostate cancer treated with definitive radiotherapy. However, there are several limitations to this study. This is a retrospective study and is subject to potential confounding from unmeasured variables. In the same vein, the retrospective nature of the study precludes the true determination of medication adherence and length of prescription usage. However, given the intended chronic (even lifelong) usage of these medications, it is safe to assume that the majority of patients with reported use of these medications, upon medication reconciliation at the time of consultation, would have durable regular usage. While the daily dosage of aspirin is largely homogenous (most patients taking 81 mg daily), the types of statins used and the dosages of statins and metformin vary, which was not included in our analysis due to the limited sample size. There is also the limitation of a small sample size for the metformin users, constituting only 12% of our study cohort, and thus, the potential statistical bias from unequal allocation of sample size for this dichotomous covariate. Despite this, metformin use was associated with PFS in all statistical analyses, including the sensitivity analyses that adjusted for obesity status and ADT duration. Finally, the follow-up duration was limited for many patients, which was attributed to the high number of deceased patients or of patients that were no longer followed up at our institutions. Because of the above limitations, larger multi-institutional cohort or randomized data from high-risk prostate cancer patients would be needed to definitively assess the impact of non-oncologic medications on prostate cancer outcomes.

## 5. Conclusions

This study presents the association of the use of non-oncologic medications, including aspirin, statins, and metformin, with progression-free survival in a large cohort of patients with high- and very high risk prostate cancer treated with definitive RT. Metformin use was associated with increased likelihood of prostate cancer disease progression within our study cohort. Further studies in larger cohorts of high-risk patients treated with definitive RT are needed to clarify the impact of these medications on prostate cancer outcomes.

## Figures and Tables

**Table 1:** Demographic and clinical characteristics.

Variable	Total (*n* = 237)
**Age** Median [IQR] (min, max)	66 [59, 73] (43, 87)
**Age** (binary)	
<65	98 (41.35%)
65+	139 (58.65%)
**Race/Ethnicity**	
Non-Hispanic White	90 (37.97%)
Non-Hispanic African American/Black	57 (24.05%)
Non-Hispanic Asian	18 (7.59%)
Hispanic	8 (3.38%)
Unknown	64 (27.00%)
**PSA at Diagnosis** (*n* = 236) Median [IQR] (min, max)	16.19 [7.69, 34.95] (1.78, 315.00)
**PSA at Diagnosis** (categorized) (*n* = 236)	
<10	79 (33.47%)
10–20	55 (23.31%)
>20	102 (43.22%)
**T Stage** (*n* = 236)	
T1	104 (44.07%)
T2	85 (36.02%)
T3	40 (16.95%)
T4	7 (2.97%)
**N Stage** (*n* = 231)	
N0	219 (94.81%)
N1	12 (5.19%)
**Gleason Score** (*n* = 236)	
6	11 (4.66%)
7	47 (19.92%)
8	90 (38.14%)
9	78 (33.05%)
10	10 (4.24%)
**ADT with Radiation**	
Yes	224 (94.51%)
No	13 (5.49%)
**ADT Duration** (*n* = 228) *	
≤6 months **	53 (23.25%)
>6 months	175 (76.75%)
**ADT Duration** (*n* = 215) *** Median [IQR] (min, max)	20 [9, 24] (1, 50)
**KPS-Stratified**	
0	174 (73.42%)
1+	14 (5.91%)
Missing	49 (20.68%)
**Aspirin Use**	
Yes	82 (34.60%)
No	155 (65.40%)
**Statin Use**	
Yes	88 (37.13%)
No	149 (62.87%)
**Metformin Use**	
Yes	29 (12.24%)
No	208 (87.76%)
**Obesity (BMI ≥ 30)**	
Yes	85 (35.86%)
No	152 (64.14%)
**Type of Definitive RT**	
Dose-escalated IMRT	222 (93.67%)
IMRT + brachytherapy boost	15 (6.33%)
**Receipt of Pelvic RT**	
Yes	144 (60.76%)
No	93 (39.24%)
**RT Prescription Dose to Prostate** (Gy) (*n* = 222) **** Median [IQR] (min, max)	78 [77.4, 79.2] (42, 80)
**Total # of RT Fractions** (*n* = 222) **** Median [IQR] (min, max)	42 [39, 44] (21, 44)

Abbreviations: PSA, prostate-specific antigen. ADT, androgen deprivation therapy. KPS, Karnofsky performance status. IQR, interquartile range. Min, minimum. Max, maximum. BMI, body mass index. IMRT, intensity-modulated radiation therapy. * Of 237 patients, 9 patients were missing ADT duration. ** Patients who received less than or equal to 6 months of ADT, including patients that did not receive ADT. *** Only including the 215 patients who received ADT with known ADT duration. **** Only including the 222 patients who received dose-escalated IMRT.

**Table 2:** Univariate Cox proportional hazards model results for progression-free survival.

Variable	Crude HR (95% CIs)	*p*-Value
**Age** (in years) 1-year increase	0.98 (0.95–1.01)	0.2468
**Age** (binary)		0.9787
<65	Reference	
≥65	1.01 (0.58–1.75)	
**Race/Ethnicity**		0.6364
Non-Hispanic White	Reference	
Non-Hispanic African American/Black	0.91 (0.46–1.80)	0.7904
Non-Hispanic Asian	0.43 (0.10–1.80)	0.2460
Hispanic	2.07 (0.48–8.92)	0.3312
Unknown	0.94 (0.47–1.90)	0.8690
**Race/Ethnicity (binary)**		0.9184
Non-Hispanic African American/Black	0.97 (0.52–1.81)	
Other	Reference	
**PSA at Diagnosis**		0.0347
<20	Reference	
≥20	1.81 (1.04–3.12)	
**T Stage**		0.0216
T1–2	Reference	
T3–4	2.00 (1.11–3.60)	
**N Stage**		0.0101
N0	Reference	
N1	3.46 (1.34–8.91)	
**Gleason Score**		0.0856
6–7	Reference	
≥8	1.95 (0.91–4.17)	
**ADT with Radiation**		0.2484
Yes	Reference	
No	0.31 (0.04–2.27)	
**ADT Duration**		0.6174
≤ 6 months	Reference	
>6 months	0.85 (0.44–1.62)	
**KPS-Stratified**		0.4074
0	Reference	
≥1	1.02 (0.31–3.30)	0.9798
Unknown	0.63 (0.32–1.25)	0.1844
**Aspirin Use**		0.5001
Yes	0.82 (0.46–1.46)	
No	Reference	
**Statin Use**		0.5733
Yes	0.84 (0.47–1.52)	
No	Reference	
**Metformin Use**		0.0443
Yes	2.20 (1.02–4.72)	
No	Reference	
**Obesity (BMI ≥ 30)**		**0.1334**
Yes	1.52 (0.88–2.64)	
No	Reference	
**Type of Definitive RT**		0.2976
Dose-escalated IMRT	Reference	
IMRT + brachytherapy boost	1.64 (0.65–4.16)	
**Receipt of Pelvic RT**		0.0747
Yes	Reference	
No	0.57 (0.30–1.06)	

Univariate Cox proportional hazards model results for progression-free survival. Abbreviations: PSA, prostate-specific antigen. ADT, androgen deprivation therapy. KPS, Karnofsky performance status. HR, hazard ratio. CI, confidence interval. BMI, body mass index. IMRT, intensity-modulated radiation therapy.

**Table 3:** Multivariable Cox proportional hazards model results for progression-free survival and aspirin use (yes vs. no).

Variable	Adjusted HR (95% CIs)	*p*-Value
**Aspirin Use**		0.7147
Yes	0.90 (0.49–1.62)	
No	Reference	
**PSA at Diagnosis**		0.0056
<20	Reference	
≥20	2.30 (1.28–4.14)	
**T Stage**		0.0329
T1–2	Reference	
T3–4	1.95 (1.06–3.61)	
**Gleason Score**		0.0077
6–7	Reference	
≥8	3.00 (1.34–6.74)	
**Race/Ethnicity**		0.4275
Non-Hispanic African American/Black	1.30 (0.68–2.51)	
Other	Reference	

Multivariable Cox proportional hazards model results for progression-free survival and aspirin use (yes vs. no). Abbreviations: PSA, prostate-specific antigen. HR, hazard ratio. CI, confidence interval.

**Table 4:** Multivariable Cox proportional hazards model results for progression-free survival and statin use (yes vs. no).

Variable	Adjusted HR (95% CIs)	*p*-Value
**Statin Use**		0.3975
Yes	0.77 (0.42–1.41)	
No	Reference	
**PSA at Diagnosis**		0.0051
<20	Reference	
≥20	2.30 (1.28–4.11)	
**T Stage**		0.0288
T1–2	Reference	
T3–4	1.98 (1.07–3.65)	
**Gleason Score**		0.0056
6–7	Reference	
≥8	3.17 (1.40–7.16)	
**Race/Ethnicity**		0.3965
Non-Hispanic African American/Black	1.33 (0.69–2.56)	
Other	Reference	

Multivariable Cox proportional hazards model results for progression-free survival and statin use (yes vs. no). Abbreviations: PSA, prostate-specific antigen. HR, hazard ratio. CI, confidence interval.

**Table 5:** Multivariable Cox proportional hazards model results for progression-free survival and metformin use (yes vs. no).

Variable	Adjusted HR (95% CIs)	*p*-Value
**Metformin Use**		0.0361
Yes	2.46 (1.06–5.72)	
No	Reference	
**PSA at Diagnosis**		0.0022
<20	Reference	
≥20	2.52 (1.40–4.55)	
**T Stage**		0.0261
T1–2	Reference	
T3–4	2.01 (1.09–3.71)	
**Gleason Score**		0.0187
6–7	Reference	
≥8	2.65 (1.18–5.99)	
**Race/Ethnicity**		0.8221
Non-Hispanic African American/Black	1.08 (0.54–2.16)	
Other	Reference	

Multivariable Cox proportional hazards model results for progression-free survival and metformin use (yes vs. no). Abbreviations: PSA, prostate-specific antigen. HR, hazard ratio. CI, confidence interval.

**Table 6:** Multivariable Cox proportional hazards model results for progression-free survival and aspirin/statin/metformin use (yes vs. no).

Variable	Adjusted HR (95% CIs)	*p*-Value
**Aspirin Use**		0.9138
Yes	1.04 (0.55–1.94)	
No	Reference	
**Statin Use**		0.2845
Yes	0.70 (0.36–1.35)	
No	Reference	
**Metformin Use**		0.0158
Yes	2.77 (1.21–6.32)	
No	Reference	
**PSA at Diagnosis**		0.0029
<20	Reference	
≥20	2.50 (1.37–4.58)	
**T Stage**		0.0220
T1–2	Reference	
T3–4	2.06 (1.11–3.82)	
**Gleason Score**		0.0127
6–7	Reference	
≥8	2.76 (1.24–6.15)	

**Table 7:** Multivariable Cox proportional hazards model results for progression-free survival and metformin use (yes vs. no) while adjusting for obesity (BMI ≥ 30).

Variable	Adjusted HR (95% CIs)	*p*-Value
**Metformin Use**		0.0476
Yes	2.30 (1.01–5.22)	
No	Reference	
**PSA at Diagnosis**		0.0012
<20	Reference	
≥20	2.73 (1.49–5.03)	
**T Stage**		0.0358
T1–2	Reference	
T3–4	1.93 (1.05–3.56)	
**Gleason Score**		0.0148
6–7	Reference	
≥8	2.70 (1.22–6.01)	
**Obesity (BMI ≥ 30)**		0.1919
Yes	1.48 (0.82–2.66)	
No	Reference	

Abbreviations: PSA, prostate-specific antigen. HR, hazard ratio. CI, confidence interval. BMI, body mass index.

**Table 8:** Multivariable Cox proportional hazards model results for progression-free survival and metformin use (yes vs. no) while adjusting for ADT duration.

Variable	Adjusted HR (95% CIs)	*p*-Value
**Metformin Use**		0.0243
Yes	2.51 (1.13–5.60)	
No	Reference	
**PSA at Diagnosis**		0.0010
<20	Reference	
≥20	2.81 (1.52–5.19)	
**T Stage**		0.0330
T1–2	Reference	
T3–4	1.98 (1.06–3.72)	
**Gleason Score**		0.0077
6–7	Reference	
≥8	3.05 (1.34–6.94)	
**ADT Duration**		0.2351
≤6 months	Reference	
>6 months	0.66 (0.33–1.31)	

Abbreviations: PSA, prostate-specific antigen. HR, hazard ratio. CI, confidence interval. ADT, androgen deprivation therapy.

## Data Availability

Research data are stored in an institutional repository and will be shared upon request to the corresponding author.
